# A brief insight into *Citrobacter* species - a growing threat to public health

**DOI:** 10.3389/frabi.2023.1276982

**Published:** 2023-12-05

**Authors:** Ishrat Jabeen, Sohidul Islam, A. K. M. Imrul Hassan, Zerin Tasnim, Sabbir R. Shuvo

**Affiliations:** Department of Biochemistry & Microbiology, North South University, Dhaka, Bangladesh

**Keywords:** *Citrobacter spp*., epidemiology, pathogenesis, multidrug resistance, treatment

## Abstract

*Citrobacter* spp. are Gram-negative, non-spore forming, rod-shaped, facultative anaerobic bacteria from the *Enterobacteriaceae* family often found in soil, sewage, sludge, water, food, and the intestinal tracts of animals and humans. Several members of *Citrobacter* spp. especially *C. freundii*, *C. koseri*, *C. braakii* are frequently detected in newborn illnesses, urinary tract infections, and patients with severe underlying conditions, including hypertension, diabetes, cancer, and respiratory infections, or those who are immunocompromised. Strains of *Citrobacter* spp. can spread vertically or horizontally from carriers or other hospital sources and thus cause nosocomial infections in hospital settings. A total of 19 *Citrobacter* genomospecies have been recognized based on genomics. It has been noted that the *Citrobacter* genus acquired antimicrobial resistance and virulence, including invasion, colonization, biofilm formation, and toxin production. The recent emergence and spread of antimicrobial resistance to β-lactams, carbapenems, fluoroquinolones, aminoglycosides, and colistin in *Citrobacter* spp. through chromosomal and plasmid-mediated resistance limits the empiric treatment options. Therefore, combination therapy involving costly and potentially hazardous antibiotics poses significant challenges in treating *Citrobacter* infections. Here we summarized the nomenclature of *Citrobacter* spp., clinical manifestations, epidemiology, pathogenesis, antibiotic resistance mechanisms, and treatments from various clinical samples. This review will expand our knowledge of the genomics and epidemiology of *Citrobacter* spp., enabling improved control of infections and the spread of these organisms.

## Introduction

*Citrobacter* spp. accounts for 3-6% of all isolates from the *Enterobacteriaceae* family, which causes nosocomial infections (Doran, 1999; Borenshtein and Schauer, 2006; Maraki et al., 2017; Rostamzad et al., 2019; Nair et al., 2020; Aguirre-Sánchez et al., 2023; Heljanko et al., 2023) *Citrobacter* spp. are found in soil, sewage sludge water, food, and the intestinal tracts of animals and humans (Cong’En et al., 2014; Forsythe et al., 2015). *Citrobacter* is considered an opportunistic nosocomial pathogen that is commonly associated with urinary tract infections (UTIs), bloodstream infections, intra-abdominal sepsis, brain abscesses, pneumonia, and other neonatal infections such as meningitis, neonatal sepsis, joint infections, or common bacteremia (Ashish et al., 2012; Maraki et al., 2017; Hawaldar and Sadhna, 2019; Räisänen et al., 2021). It is evident that the two prominent opportunistic pathogens, *C. koseri* and *C. freundii*, account for most of the *Citrobacter* infections where more than 80% of patients were found to have underlying medical conditions including diabetes, cardiovascular disease, renal disease, leukemia, neurologic disease, or abnormalities of the urinary tracts (Mohanty et al., 2007; Liu et al., 2018c; Chen and Ji, 2019; Lalaoui et al., 2019; Lee et al., 2019; Khan et al., 2020; Dominguez Céspedes and Céspedes Fonseca, 2022; Hua et al., 2022; Ramachandran et al., 2022).

*Citrobacter* spp., mainly *C. freundii*, has started to cause various diseases, and they are also becoming increasingly resistant to several types of antibiotics (Liu et al., 2017). *C. freundii* is often considered more resistant than *C. koseri* to β-lactam antibiotics, including amoxicillin, amoxicillin-clavulanate, ampicillin, first- and second-generation cephalosporins. The incidence of antibiotic-resistant *Citrobacter* isolates has been reported increasingly worldwide(Osei Sekyere and Reta, 2021). Despite advances in diagnostic methods and antibiotic therapy, *Citrobacter* infections are considered fatal, with case-fatality rates of 30% and death rates of 33-48% in neonates (Pepperell et al., 2002; Hewitt et al., 2021). Surviving infants can experience significant central nervous system (CNS) damage, including severe intellectual disability, hemiplegia, and seizures. Phylogenomic research revealed that, regardless of boundaries, a single strain gradually evolved during diffusion from host to host (Osei Sekyere and Reta, 2021).

This review provides insight into the nomenclature, clinical manifestations, pathogenesis, antibiotic resistance mechanism, and possible treatment options for infections caused by clinical strains of *Citrobacter* spp.

## Classification and nomenclature

In 1928, Braak identified two bacterial strains that could convert glycerol to trimethylene glycol without oxygen. The strains were named “*Bacterium freundii*” in remembrance of Freund’s 1881 discovery that found trimethylene glycol was a by-product of glycerol fermentation (Braak, 1928). Later, Werkman and Gillen proposed the genus *Citrobacter* in 1932 (Werkman and Gillen, 1932). As the name implies, members of the genus *Citrobacter* utilize citrate as the primary carbon source and produce acid and gas due to the fermentation of glucose and numerous other carbohydrates (Brenner et al., 1999). However, in the following years, many synonyms have been proposed to describe such organisms, including *Escherichia freundii* and *Salmonella ballerup* (Fritsche, 1964; Schoch et al., 2020). But finally, in 1958, the International Subcommittee on the Taxonomy of the *Enterobacteriaceae* agreed to recognize the name “*Citrobacter freundii*” for this heterogenous group of bacteria, followed by the recognition of another two groups of bacteria similar to *C. freundii* The first group has been named as “*C. koseri,”* “*C. diversus*” or “*Levinea malonatica*” and the second group has been designated as “*L. amalonaticus*”. In 1993, *C. diversus* was formally named as *C. koseri*, granted by the Judicial Commission of the International Committee on Systematic Bacteriology (Borenshtein and Schauer, 2006; Schoch et al., 2020).

**Figure 1:**
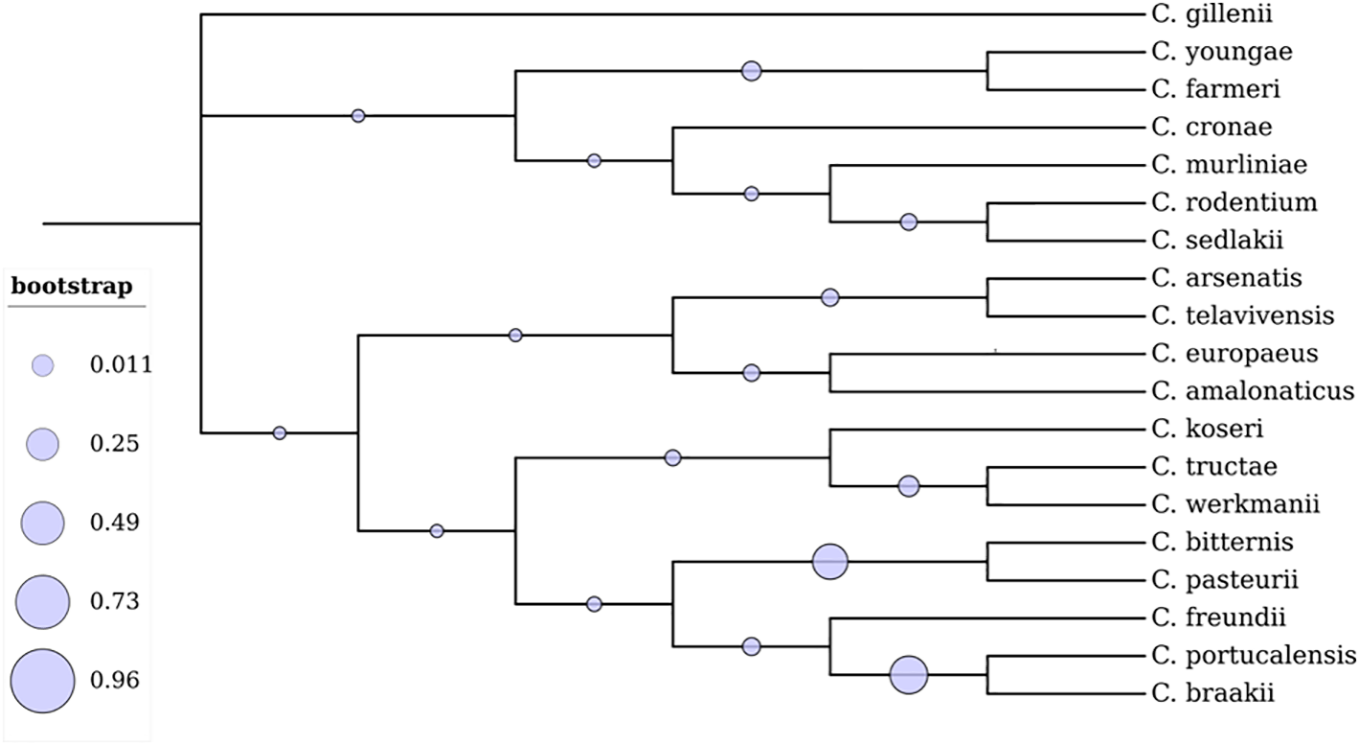
Phylogenetic analysis of 19 *Citrobacter* spp. The branches display the percentage of trees where related groups of species clustered together. The evolutionary tree was constructed using the Maximum Likelihood technique in the MEGA X software (Kumar et al., 2018). The tree was visualized using Interactive Tree of Life (iTOL).

Bacterial classification is essential for microbial diversity, diagnostic purposes, serotyping, and scientific studies. *Citrobacter* isolates have been identified based on biochemical and carbon source utilization tests, and detailed biotypes have been reported by Brenner et al. (Brenner et al., 1999). Taxonomically it is evident that the genus *Citrobacter* is most closely related to *Salmonella* and *Escherichia coli* (Katzenellenbogen et al., 2017; Schoch et al., 2020). A total of 19 *Citrobacter* genomospecies have been recognized based on DNA relatedness to date (**Table 1**; **Figure 1**) (Borenshtein and Schauer, 2006; Oberhettinger et al., 2020; Ribeiro et al., 2021; Wang et al., 2021). Additionally, based on the lipopolysaccharide (LPS) O antigen, a total of 43 *Citrobacter* O-serogroups have been elucidated and also a total of 20 chemo groups have been classified according to the sugar composition of their lipopolysaccharides (LPS) (Keleti et al., 1971; Gross and Rowe, 1974; Knirel et al., 2002). Such OPS is crucial for serological classification of bacterial strains cross-reactivity between *Citrobacter* and other *Enterobacteriaceae* spp. This, in turn, will provide further insights in understanding the mechanism of antibiotic susceptibility patterns of *Citrobacter spp*, to cause infections (Katzenellenbogen et al., 2017).

**Table 1:** 19 *Citrobacter* spp. and their associated NCBI accession number of 16S rRNA gene sequences and Reference Genome ID.

SI	Name	16S rRNA Nucleotide Accession Number	Reference Genome ID
1	*C. amalonaticus*	KM515966	ASM155893v2
2	*C. arsenatis*	MK262983	ASM435384v1
3	*C. bitternis*	OK035353	NA
4	*C. braakii*	KM515967	ASM964893v1
5	*C. cronae*	MN548424	ASM1689368v1
6	*C. europaeus*	LT615140	ASM379537v1
7	*C. farmeri*	KM515968	ASM393820v1
8	*C. freundii*	KM515969	ASM381234v1
9	*C. gillenii*	KM515970	ASM342960v1
10	*C. koseri*	KP728117	ASM1804v1
11	*C. murliniae*	KY178281	ASM480112v1
12	*C. pasteurii*	KP057683	ASM1904776v1
13	*C. portucalensis*	OQ073593	ASM869360v1
14	*C. rodentium*	KM515972	ASM2127898v1
15	*C. sedlakii*	KM515973	ASM1812842v1
16	*C. telavivensis*	MN603664	ASM929566v1
17	*C. tructae*	KM515975	ASM468434v1
18	*C. werkmanii*	KM515974	ASM869364v1
19	*C. youngae*	KM515975	ASM3029458v1

NA, Not available in NCBI.

## Clinical manifestations

A number of *Citrobacter* spp. are opportunistic pathogens known to be the causative agent of severe infections, sepsis, respiratory infections, UTIs, keratitis, and meningitis, particularly in high-risk populations like infants and immunocompromised adults (Doran, 1999; dos Santos et al., 2015; Garcia et al., 2016; Jiménez et al., 2017; Emery et al., 2020; Urbinati et al., 2023). Furthermore, skin infections like folliculitis, cellulitis, hives, ulcers, and necrotizing fasciitis were also associated with *Citrobacter* spp. (Mohanty et al., 2007; Raia et al., 2015; Licata et al., 2021; Moussa et al., 2023). Among the *Citrobacter* spp. *C. freundii* and *C. koseri* are frequently isolated mostly from UTIs in addition to surgical wounds, respiratory infections, and gastrointestinal infections (Mohanty et al., 2007; Metri et al., 2013; Gajdács and Urbán, 2019; Hossain et al., 2021). The clinical manifestation associated with *Citrobacter* spp. is addressed below in detail.

### Bacteremia

Bacteremia caused by *Citrobacter* spp. can be nosocomial or community-acquired (Doran, 1999; Lai et al., 2010; Dziri et al., 2022). Also, infections caused by *Citrobacter* spp. are commonly linked to polymicrobial bacteremia (Shih et al., 1996; Hashimoto et al., 2021). Diabetics, hypertension, cancer, and liver cirrhosis are the frequent underlying medical conditions associated with *Citrobacter* spp. infections (Lai et al., 2010; Hashimoto et al., 2021; Casas-Martínez et al., 2023). Therefore, the primary sites of infection and prognosis of *Citrobacter* bacteremia are still under investigation.

The frequent initial symptom of bacteremia caused by *Citrobacter* spp. in patients is fever with shivering. However, few individuals experience hypothermia or average body temperature (Hashimoto et al., 2021; Hua et al., 2022). A small group of patients also experienced hypotension, oliguria, and impaired mental status. Jaundice, ileus, abdominal pain, and/or gastrointestinal bleeding are other indicators of bacteremia caused by the *Citrobacter* spp. (Lai et al., 2010; Hewitt et al., 2021). Numerous significant health problems from *Citrobacter* bacteremia, including liver dysfunction, respiratory failure, renal dysfunction, and thrombocytopenia, are also reported (Shih et al., 1996). Additionally, *C. koseri* has been recently isolated from transfusion-related bacteremia and keratitis (Emery et al., 2020; Urbinati et al., 2023).

### Meningitis

The role of *Citrobacter* spp. in infants and adult meningitis following central nervous system (CNS) abscesses is well documented (Doran, 1999; Cuadros et al., 2013; Chen and Ji, 2019; Hewitt et al., 2021; Bonasoni et al., 2022). *C. koseri* has a significant preference for the CNS in the first two months of life, whereas it also frequently causes meningitis and brain abscesses, brain adhesions, encephalitis, and pneumocephalus (Doran, 1999; Vaz Marecos et al., 2012; Cuadros et al., 2013; Hewitt et al., 2021; Bonasoni et al., 2022). Additionally, strains of *C. freundii* can cause meningitis in adults and infants (Plakkal et al., 2013; Lien et al., 2018).

Infections caused by *Citrobacter* spp. spread horizontally through hospital settings or vertically through mother-to-child contact; however, the source of the infection is primarily unclear (Doran, 1999; Plakkal et al., 2013; Hewitt et al., 2021). In infants, if the symptoms appear in the first days of life, vertical transmission is most likely the cause of infection. However, isolation of the pathogens from the mother is rare (Bonasoni et al., 2022). *C. koseri* infection in newborns can be divided into early onset (5-12 days of age) and late-onset (4-5 weeks of age) (Doran, 1999). Compared to 1% of cases from other sources, brain abscesses occur in about 80% of cases of *C. koseri* meningitis. The mortality rate for *C. koseri* meningitis is around 30%, and more than 80% of such cases also have neurological sequelae (Bonasoni et al., 2022). Fever, altered consciousness, and headache were the typical clinical manifestations of meningitis; therefore, computerized tomography (CT) scans and cerebrospinal fluid cultures were necessary to detect meningitis and its causative agent (Liu et al., 2015a; Lien et al., 2018). Furthermore, it has also been reported the involvement of *C. braakii*, *C. amalonaticus*, and *C. sedlakii* in meningitis (Lai et al., 2010; Hirai et al., 2016; Tripathi et al., 2020).

### Epidemiology

Recently, a genome-wide epidemiological investigation of 686 *Citrobacter* strains from 67 countries was reported (Osei Sekyere and Reta, 2021). In this analysis, only *C. freundii* had a multi-locus sequence typing (MLST) scheme among the studied strains. In that study, *C. freundii* had 84 distinct clones or sequence types (STs). The study reported the most prevalent clones were ST100, ST22, ST62, ST11, ST299, ST8, ST114, and ST98 (Osei Sekyere and Reta, 2021). They also observed that three clades of *Citrobacter* spp. are predominant in different parts of the world. In Clade A, *C. freundii* was primarily distributed in Europe, North America, and South East Asia, *C. koseri* is present only in the USA, and *C. amalonaticus* was observed in North America and South Korea. In clade B, *C. freundii* was identified worldwide; *C. koseri* was identified in the USA, Europe, China, Malaysia, and Malawi, whereas *C. amalonaticus* was identified in France, Malaysia, and the USA. Clade C only contained *C. amalonaticus*, found in Malaysia, Switzerland, and the USA (Osei Sekyere and Reta, 2021).

Another six-year epidemiological investigation on short-term bloodstream infections associated with peripheral venous catheters was conducted in 14 Middle Eastern nations, and the results showed that *Citrobacter* spp. was responsible for 1% of the infections(Rosenthal et al., 2020). Additionally, a meta-analysis that mostly comprised publications published in Iran between 2012 and 2018 found that the antibiotic sensitivity patterns of *C. freundii* varied by region (Rostamzad et al., 2019). This review also includes major investigations of *Citrobacter* spp. from various clinical samples from different countries (**Table 2**).

**Table 2:** A list of major studies of *Citrobacter* spp. isolated from various clinical samples worldwide.

SL	Year of sample isolation	Type of Sample	Type of *Citrobacter* spp.	Country of origin	References
**1**	2019-2020	Urine	*Citrobacter* spp. *(2)*	Bangladesh	(Farjana et al., 2021)
**2**	2019-2020	Urine, stool, wound swab, pus, blood, sputum	*C. freundii (27)*	Bangladesh	(Rahman et al., 2022)
**3**	Not mention	Urine	*Citrobacter* spp. *(3)*	Bangladesh	(Hossain et al., 2021)
**4**	2007-2011	Diarrheal patients	*C. freundii (13), C. braakii (8), C. youngae (41)*	China	(Liu et al., 2017)
**5**	2014-2016	Diarrheal patients & healthy person	*C. freundii (82)*	China	(Liu et al., 2018c)
**6**	2014-2018	UTI, sputum, bile, secretion, blood	*C. freundii (26), C. braakii (6), C. koseri (14)*	China	(Liu et al., 2021b)
**7**	2016-2017	Diarrheal patients	*C. freundii (30), C. braakii (8), C. youngae (12)*	China	(Liu et al., 2020)
**8**	2011	Wound	*Citrobacter* spp. *(18)*	Ethiopia	(Godebo et al., 2013)
**9**	2019	Urine, sputum, wound	*Citrobacter* spp. *(5)*	Ethiopia	(Tadesse et al., 2022)
**10**	2007	Urine, wound,	*Citrobacter* spp. *(45)*	France	(Lavigne et al., 2007)
**11**	2010-2015	Not Mentioned	*C. freundii (172), C. koseri (166), C. braakii (34), C. amalonaticus (6), C. youngae (6), C. sedlakii (1)*	Greece	(Maraki et al., 2017)
**12**	1979	Diarrheal patients	*C. freundii (4)*	India	(Pardia et al., 1980)
**13**	2004	Urine, respiratory tract, blood, pus, sterile body fluid	*C. freundii (20), C. koseri (185)*	India	(Mohanty et al., 2007)
**14**	2018	Urine, pus, vaginal swab	*Citrobacter* spp. *(12)*	India	(Hawaldar and Sadhna, 2019)
**15**	2009-2010	Urine	*C. koseri (55)*	India	(Metri et al., 2013)
**16**	2009-2010	Urine, sputum, nasal and throat swabs	*Citrobacter* spp. *(348)*	Japan	(Kanamori et al., 2011)
**17**	2003	Clinical samples (did not mention type)	*C. freundii (21)*	Korea	(Kim and Lim, 2005)
**18**	2007-2017	Intra-abdominal, urine, catheter, soft tissue	*C. freundii (29), C. koseri (5), C. braakii (6), C. amalonaticus (2), C. youngae (1)*	Korea	(Lee et al., 2019)
**19**	2009-2014	Intra-abdominal, urine, catheter, soft tissue	*C. freundii (36)*	Taiwan	(Liu et al., 2018b)
**20**	2010	Baby cots, incubators, face masks, nasal prongs, stethoscopes	*Citrobacter spp (29)*	Nepal	(Khadka et al., 2011)
**21**	2000-2005	Urine, bloodstream infection, skin, soft tissue	*C. koseri (428)*	North America, Latin America, Asia Pacific, Europe	(Castanheira et al., 2009)
**22**	2016-2017	Urine, pus, wound	*C. freundii (130)*	Pakistan	(Khan et al., 2020)
**23**	2013-2014	Urine	*C. freundii (22)*	Sierra Leone	(Leski et al., 2016)
**24**	1961	Urine	*Citrobacter* spp. *(38)*	UK	(Whitby and Muir, 1961)

The number in () indicates number of samples.

Recently, carbapenem-resistant *Citrobacter* spp., especially *C. freundii* strains, were frequently isolated from Europe (Arana et al., 2017; Räisänen et al., 2021; Yao et al., 2021). In Spain, from 2013-2015, an increasing number (53%) of carbapenemase-producing *Citrobacter* spp. were isolated (Arana et al., 2017). During the years 2017, 2018, and 2019, *Citrobacter* spp. constituted 10%, 17%, and 14% of the total carbapenemase-producing strains identified in Germany, respectively (Yao et al., 2021). Furthermore, genomic and epidemiologic studies performed between 2000-2018 in the USA found carbapenem-resistant *Citrobacter* spp. increased from 4% to 10% (Babiker et al., 2020).

## Pathogenesis

The majority of the infections caused by *Citrobacter* spp. are primarily associated with *C. koseri* and *C. freundii* isolated from human clinical specimens (Samonis et al., 2009). However, only experimental and *in silico* serotyping systems for *Citrobacter* detection have been developed to manage infections caused by it (Qian et al., 2018). It has been proposed that the affinity of *C. koseri* for nerve tissues and its propensity to induce meningitis and abscesses were related to a unique 32 kilodalton (kDa) outer-membrane protein (Southern and Bagby, 1977; Kline et al., 1988; Li et al., 1990).

In *C. koseri*, major virulence factors were associated with flagellar apparatus biosynthesis and iron uptake. *C. koseri* was found to have a High Pathogenicity Island (HPI) gene cluster, similar to a highly pathogenic *Yersinia* strain, enabling iron uptake in iron-deficient environments. The presence of such HPI could explain the remarkable pathogenic effects of *C. koseri* on the CNS. In contrast, *C. freundii* and *C. braakii* contained genes encoding the VI capsule polysaccharide. This VI capsule polysaccharide contributes to evading host defenses by *Salmonella typhi*, possibly leading to the higher pathogenic potential of *C. freundii* and *C. braakii* (Yuan et al., 2019). Furthermore, flagellar apparatus, tad pilus, and type IV pilus were unique to *Citrobacter* spp., whereas types II, III, IV, V, & VI secretion systems were found in some, but not all strains. Also, *C. koseri* lacked several of these secretion systems and the tad pilus, which are thought to be critical for colonizing human environments. The study also revealed three classes of Type VI secretion system (T6SS) genes, acquired through horizontal gene transfer, with distinct functions in biofilm formation (T6SS-1), colonization, survival, or invasion (T6SS-2), and antibacterial activity (T6SS-3) of *Citrobacter* spp. In *C. koseri* strains, only T6SS-2 genes were identified (Yuan et al., 2019).

The mechanism of infection and pathogenesis by *C. freundii* is known to occur through the T6SS and its effectors Hemolysin-coregulated *protein (*Hcp) family proteins, comprises Hcp-1 and Hcp-2, which are localized in the bacterial outer membrane and prevent phagocytosis by macrophage along with Vgr family orthologs (Zheng and Leung, 2007; Liu et al., 2015b; Aubert et al., 2016; Liu et al., 2021a). The T6SS effector Hcp-2 triggers IL-1β secretion *via* Nucleotide Oligomerization Domain - Like Receptor Family, Pyrin Domain Containing-3 (NLRP3)-dependent activation of caspase 1. Caspase 1 cleaves the gasdermin–N domain (GSDMD) to mediate the pyroptosis of macrophages (Yuan et al., 2019).

Several *C. freundii* strains were found to carry virulence factors including Shiga-like toxins and heat-stable toxins or virulence islands, thus associated with diarrhoea and food poisoning in humans. Shiga-like Toxin -II (SLT-II) has been reported in seven *C. freundii* strains, which had the same degree of cytotoxicity as the *E. coli* SLT-IIvhc control strain (Liu et al., 2020).

Another study revealed the presence of 3 virulence genes (*hcp, msgA*, and, *rtx*) contributed by 152 intact prophages associated with *C. freundii* strains (Jabeen et al., 2023). The most predominantly distributed *MsgA* plays a role in the biofilm formation and antibiotic resistance in *Bacillus subtilis* and *Staphylococcus aureus* (Branda et al., 2004; Lindsay, 2014). Thus, it might be assumed to contribute to biofilm formation and antibiotic resistance in *C. freundii*. The same study also identified the *RTX* as the second highly distributed virulence gene among those intact prophages, which has not been identified in *Citrobacter* spp. yet. The presence of such protein with hemolytic activity in some prophages may confer new virulence factors and/or antibiotic resistance genes required for bacterial pathogenesis and beneficial traits like increased fitness to the host (Boyd, 2012; Bobay et al., 2014).

**Figure 2:**
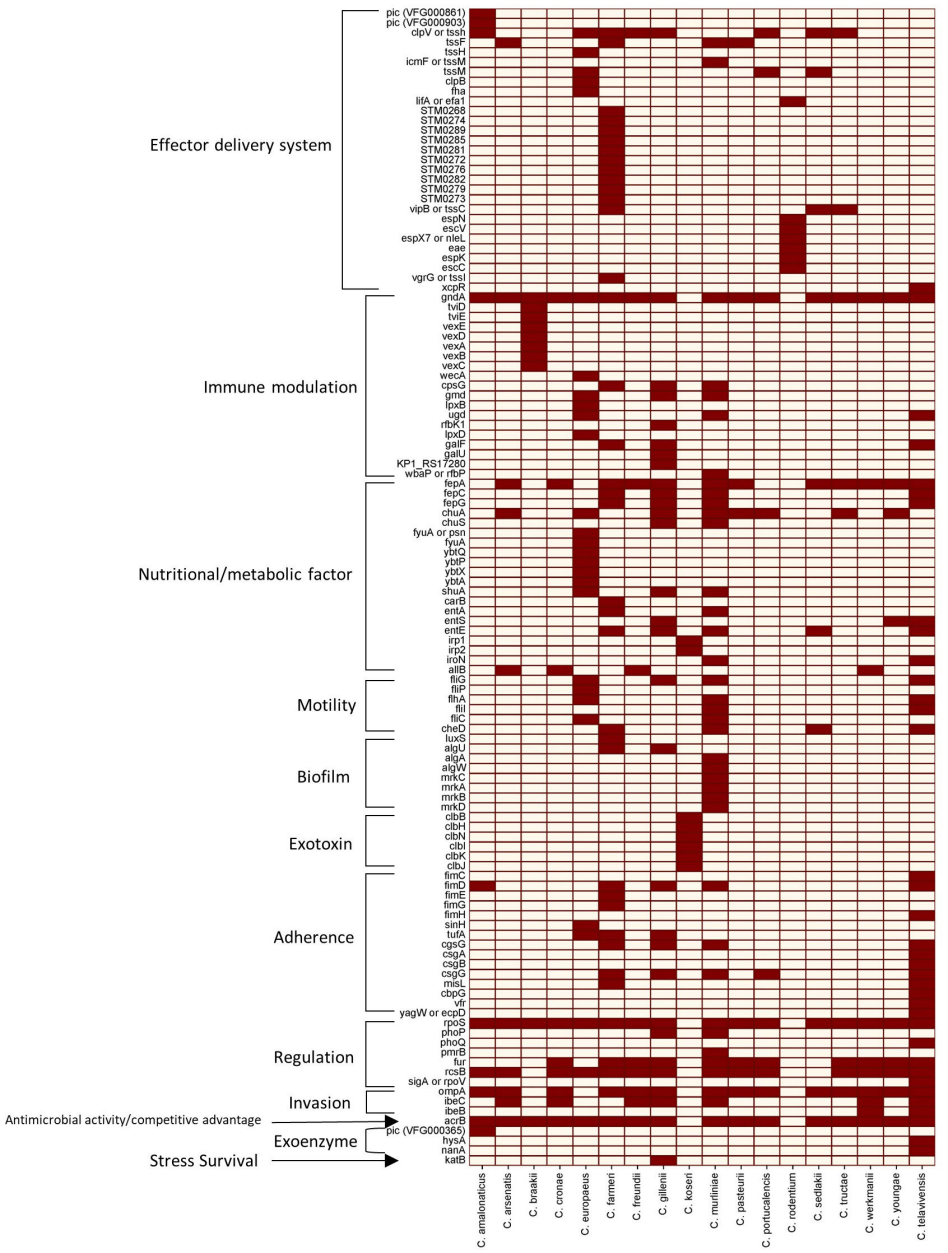
Heat map of the 118 virulence proteins across 18 different *Citrobacter* spp. studied. Dark brown squares denote the presence of the genes, and grey squares denote the absence of the genes listed.

The distribution of virulence proteins across the reference genomes of 18 different *Citrobacter* spp. have been investigated against the virulence factor data base (VFDB) (Liu et al., 2022). The genome IDs of the total of 18 *Citrobacter* spp. has been derived from National Center for Biotechnology Information (NCBI) as shown in **Table 1**.The heat map, generated using SRplot (https://www.bioinformatics.com.cn/en) (**Figure 2**), analysis of a total of 118 virulence proteins reveals that all the *Citrobacter* spp. examined are associated with virulence traits conferring immunomodulation, regulation and antimicrobial activity with the exception in *C. koseri*, and *C. rodentium* strains. Additionally, 78% of the studied *Citrobacter* spp. are found to have virulence factors associated to regulation and invasion.

## Antibiotic resistance

**Figure 3:**
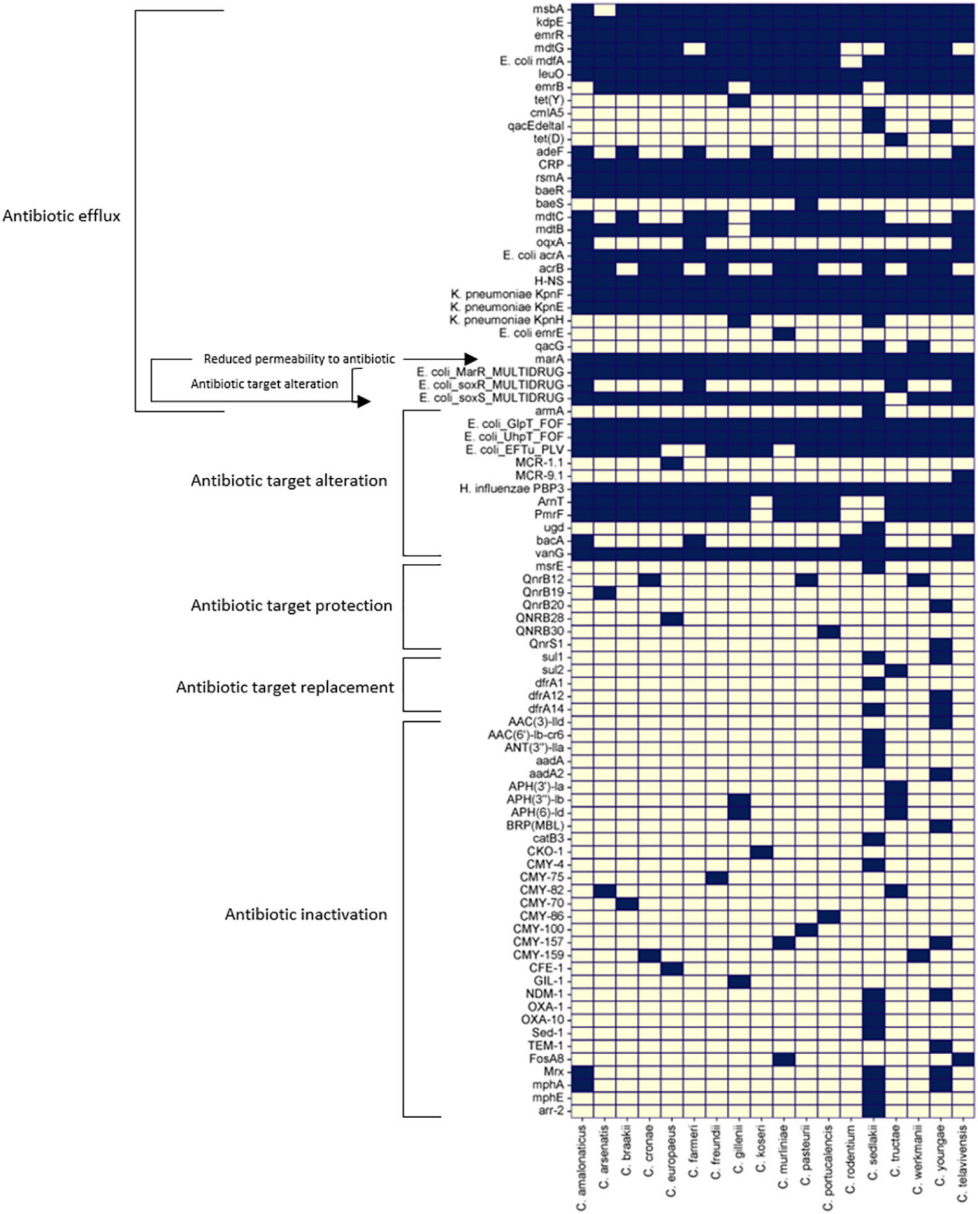
Heat map of 86 antibiotic resistant genes across the reference genomes of 18 different *Citrobacter* spp. studied were predicted using CARD database (Alcock et al., 2020) Dark blue squares denote the presence of the genes and grey squares denote the absence of the genes listed.

Antibiotic resistance in *Citrobacter* spp. become a growing public health concern. *Citrobacter* spp. possess several antibiotic-resistant determinants encoded either in the plasmid or chromosome (Pepperell et al., 2002; Poire et al., 2011; Liu et al., 2017; Liu et al., 2018b; Liu et al., 2020; Shinu, 2022; Huang et al., 2023). They also have an increased propensity to take up genetic material conferring antibiotic resistance from other related or unrelated species (Frenk et al., 2021). The presence of a total of 86 naturally occurring antibiotic-resistant determinant genes identified in the reference genomes of 18 *Citrobacter* spp. is shown in a heatmap according to the Comprehensive Antibiotic Resistance Database (CARD) Resistance Gene Identifier (**Figure 3**). They mostly rely on antibiotic efflux and target alteration to confer resistance to the antibiotics they are exposed to, except for *C. sedlakii* and *C. youngae*, which carry numerous other antibiotic-resistant determinants.

In this section of the review, we have summarized recent antibiotic resistance trends and mechanisms of *Citrobacter* spp.

There are several ways through which *Citrobacter* spp. can be resistant to fluoroquinolone antibiotics. Several studies have reported mutations in quinolone resistance-determining regions (QRDR) in *Citrobacter* spp., especially *C. freundii*, isolated from different sources and geographic regions (Weigel et al., 1998; Minarini, 2012; Kotb et al., 2019). For example, a common mutation in codon 59 (T59I) of *gyrA* was found in quinolone-resistant *C. freundii* isolates from China (Liu et al., 2021b). Besides, mutations such as S83L and D87N in *gyrA* and S80I in *parC* have also been detected in *C. freundii* isolates from various countries (Weigel et al., 1998; Minarini, 2012; Kotb et al., 2019).

However, some *Citrobacter* spp. have acquired resistance to quinolones through plasmids. One of the mechanisms of plasmid-mediated quinolone resistance (PMQR) in *Citrobacter* spp. is the expression of *qnr* genes. The *qnr* encodes the pentapeptide repeat family protein that binds to and protects DNA gyrase and topoisomerase IV from quinolone inhibition. The *qnrB* is the most common and diverse among *Citrobacter* spp. out of six qnr families (*qnrA*, *qnrB*, *qnrC*, *qnrD*, *qnrS*, and *qnrVC*) (Park et al., 2007; Jacoby et al., 2011). In *Citrobacter* spp., almost two-thirds of the alleles have been reported as *qnrB*, and several were shown to be located on the chromosome (Jacoby and Hooper, 2013). Second to *qnrB*, it has also been reported that *Citrobacter* spp. harbour *qnrS* in isolates producing *bla_CTX-M-2_* or *bla_CTX-M-15_* genes (Kanamori et al., 2011; Liu et al., 2018c). The *qnrS* are usually associated with transposable elements on plasmids; are often incorporated into sul1-type integrons, which are genetic platforms for antibiotic resistance gene capture and expression (Jacoby et al., 2014).

Furthermore, PMQR in *Citrobacter* spp. is the modification of quinolones by a variant of the aminoglycoside acetyltransferase AAC(6’)-Ib. This enzyme can acetylate quinolones with an amino nitrogen target, such as ciprofloxacin and norfloxacin, and reduce their antibacterial activity. This variant AAC(6’)-Ib-cr has two amino acid substitutions (W102R and D179Y) that enhance its ability to acetylate quinolones (Robicsek et al., 2006). The *aac(6’)-Ib-cr* gene is often found on plasmids with other resistance genes, such as *bla_CTX-M_* (Perilli et al., 2009; Liu et al., 2011; Goudarzi and Fazeli, 2017).

Lastly, in *Citrobacter* spp., PMQR increases quinolone efflux *via* plasmid-encoded pumps OqxAB and QepAB, which belong to the major facilitator superfamily (MFS) and the resistance-nodulation-cell division (RND) family, respectively. They can extrude many substrates, including quinolones, from the bacterial cell. The *qepAB* and *oqxAB* genes are also frequently linked with other resistance genes on plasmids or integrons (Jacoby et al., 2014).

PMQR in *Citrobacter* spp. poses a serious threat to public health, as it can compromise the efficacy of quinolones, which are important drugs for treating infections caused by MDR bacteria. Moreover, PMQR can facilitate the selection of higher-level resistance by chromosomal mutations in DNA gyrase and topoisomerase IV (Strahilevitz et al., 2009). The prevalence and diversity of quinolone resistance in *Citrobacter* spp. is an emerging problem in this genus and warrants further surveillance and molecular characterization.

The recent rise of carbapenem-resistant *Citrobacter* spp. cause severe public health concerns worldwide. Studies on carbapenem-resistant *Citrobacter* spp. found that the Inc family of plasmids was the primary group that carries genes for carbapenem resistance (Cao et al., 2021; Dziri et al., 2022; Ju et al., 2022); and was one of the main reasons for the rapid dissemination of carbapenem-resistant *Citrobacter* spp. around the world.

A report of an extremely drug-resistant strain of *C. freundii* was identified in a patient from India (Poire et al., 2011). This strain produced the NDM-1 enzyme, which is known to confer resistance to carbapenem antibiotics. In addition to the NDM-1 enzyme, the strain also produced several other ESBL-producing genes, including *bla_OXA-1_, bla_OXA-9_, bla_OXA-10_, bla_OXA181_, bla_TEM-1_, bla_VIM-4_, and bla_CMY_* genes indicating the potential for rapid dissemination of multidrug resistance among bacterial populations (Poire et al., 2011). The IncX3 plasmid pZY-NDM1 was also reported by another study harboring the *bla_NDM-1_* gene in a *C. portucalensis clinical* strain (Cao et al., 2021). The co-occurrence of multiple antibiotic resistance determinant genes greatly limits the treatment options for infections. In a UTI isolate, *C. amalonaticus* was found resistant to carbapenems and colistin conferred by *bla_NDM-1_* and *mcr-1.5*, respectively. *bla_NDM-1_* and *mcr-1.5* co-occurred in separate plasmids of type 1 IncC2 and incompatibility group Incl2, respectively. The isolate showed reduced susceptibility to carbapenems, 3^rd^ and 4^th^-generation cephalosporins, aminoglycoside, trimethoprim-sulfamethoxazole, and colistin (Faccone et al., 2019). Moreover, the *bla_NDM-1_*, found to coexist with *armA* in the *C. sedlakii* strain, isolated from the same patient, is another example of conferring resistance to a broad range of antibiotics, including carbapenems and aminoglycosides *via* horizontal transfer (Moser et al., 2021). Co-existence of *bla_NDM-1_* with *bla_SHV-12_* on the same transferrable IncX3 plasmid pZY-NDM1 in *C. freundii* and co-production of *NDM-1* and *OXA-10* in *C. braakii* isolate on different plasmids was also reported (Zhang et al., 2021; Han et al., 2022).

Two decades ago, the most prevalent isolate found was *C. freundii*, followed by *C*. *werkmanii*, *C. koseri*, and *C. farmeri*, where most of the *C. freundii* isolates harboring *bla_KPC-3_ genes*, followed by a few *bla_KPC-2_ and bla_NDM-1_* genes (Babiker et al., 2020). This study also reported the presence of the *bla_KPC-3_* gene in *C. farmeri* and *C. werkmanii*. A similar study detected plasmid-borne *bla_NDM-1_*, *bla_CMY-48_*, *bla_CTX-M-15_*, *bla_OXA-10_*, *bla_OXA-1_*, bla*_TEM-1B_* in a South African extensively drug-resistant (XDR) strain of *C. freundii* (Ramsamy et al., 2020). Unlike efflux genes, most resistant determining genes are in plasmids in this strain. *Citrobacter* spp. isolates have developed resistance to carbapenems due to the spread of carbapenemases such as NDM, VIM-1, OXA-48, and VIM-2. Carbapenem-resistant *C. freundii* carrying the *bla_NDM-1_* gene has been increasingly reported in countries such as China, India, Denmark, and South Africa (Yang et al., 2018). In contrast, *C. freundii* strains that are positive for VIM-1 and VIM-2 have been documented in Europe (Gaibani et al., 2013; Porres-Osante et al., 2014; Santos et al., 2017).

Carbapenem-resistant *Citrobacter* isolates are a diverse group of bacteria that can acquire carbapenem resistance through horizontal gene transfer. They do not usually form a single clonal complex, but sometimes isolates from different hospitals can be genetically similar, suggesting the potential for clonal spread (Yao et al., 2021). Carbapenem resistance can also be acquired through chromosomal mutations. In a *Citrobacter freundii* strain, carbapenem resistance was conferred by *marA*, *soxS*, and mutations in penicillin-binding proteins (PBP3) (Yap et al., 2020).

The prevalence of various aminoglycoside-modifying enzymes (AME) in *Citrobacter* spp. depending on the geographic region and the type of infection. Among the AMEs, the most prevalent was aminoglycoside-N-acetyltransferases (AACs). Since its discovery, the most common AAC found globally in several studies is aac(6’)-ib-cr, followed by aac(6’)-II and aac(3’)-II enzymes in *Citrobacter* spp. (Jiang et al., 2019; Zhou et al., 2019; Babiker et al., 2020; Ramsamy et al., 2020; Cao et al., 2021; Yao et al., 2021; Zhang et al., 2021; Han et al., 2022). AAC(6’)-I enzymes are highly active in inactivating amikacin and gentamicin (C1a & C2), whereas AAC(6’)-II enzymes do not modify amikacin but modify all three types of gentamicin (C1, C1a, and C2) (Woloj et al., 1986; Rather et al., 1992; Shaw et al., 1993). Furthermore, AAC(3’)-II enzymes are active against gentamicin, netilmicin, tobramycin, sisomicin, 2′-N-ethylnetilmicin, 6′-N-ethylnetilmicin, and dibekacin (Shaw et al., 1993).

After AACs, the 2^nd^ most prevalent AME is the aminoglycoside-O-phosphotransferases (APH), mainly *aph(3′′)-Ib*, *aph(6)-Id* followed by *aph(3’)-Ia* (Babiker et al., 2020; Ramsamy et al., 2020; Cao et al., 2021; Zhang et al., 2021). APH(3′′) confers resistance against streptomycin, whereas the APH(3′) group of enzymes is responsible for the resistance profile against kanamycin, neomycin, paromomycin, ribostamycin, lividomycin and is usually found in large plasmids or as part of transposon (Vakulenko and Mobashery, 2003).

The least prevalent AME in *Citrobacter* spp. belongs to aminoglycoside-O-necleotidyl transferases (ANT), and only a few ANT genes, *ant(2”)-Ia*, also known as *aadB*, was found in an IncA/C2 plasmid (Babiker et al., 2020). These genes conferred resistance to gentamicin, tobramycin, dibekacin, sisomicin, and kanamycin (Vakulenko and Mobashery, 2003). A number of studies reported that few ANT(3”) enzymes, encoded by *aadA* (*aadA1*, *aadA2*, *aadA16*), conferred resistance to spectinomycin and streptomycin in *Citrobacter* spp. (Babiker et al., 2020; Ramsamy et al., 2020; Moser et al., 2021; Han et al., 2022; Luo et al., 2022). The presence of streptomycin inactivating genes (*strA, strB*) was also reported in *Citrobacter* spp. (Yao et al., 2021; Luo et al., 2022).

In two studies, aminoglycoside resistance caused by target methylation was observed in *C. sedlakii* and *C. portucalensis*. This resistance was due to the presence of the *armA* gene in *C. sedlakii* and both *armA and rmtC* genes in *C. portucalensis* (Moser et al., 2021; Luo et al., 2022). In *C. portucalensis*, *armA* were coharbored with *aacA4cr*, *aphA1 bla_SHV-12_* gene on a separate IncC group plasmid, whereas *rmtC* gene was coharboured with IncFII : FIB plasmid-borne *bla_NDM-1_* gene (Luo et al., 2022). In addition, the involvement of efflux genes, *baeR* and *kdpE*, was reported in a clinical MDR *C. freundii* strain showing resistance against gentamicin and tobramycin (Yap et al., 2020).

The emergence and dissemination of plasmid-mediated colistin resistance in *Citrobacter* spp., particularly mediated by the mobilized colistin resistance *(mcr) genes*, is a growing concern. Several variants of *mcr* have been recently reported in many spp. of *Citrobacter*, including *mcr-1* in *C. freundii* (Li et al., 2017; Yan Hu et al., 2017) and *mcr-1* in *C. braakii* (Sennati et al., 2017; Liu et al., 2018a), and *mcr-1.5* in *C. amalonaticus* (Faccone et al., 2019), *mcr-3.5* in *C. sedlakii* and *C. amalonaticus* (Phuadraksa et al., 2023), *mcr-9* (Bitar et al., 2020) in *C. freundii* and, chromosomal *mcr-9 in C. telavivensis* (Ribeiro et al., 2021). In *Citrobacter* spp., *mcr* can co-occur with genes conferring resistance to β-lactams (Faccone et al., 2019) and aminoglycosides (Ju et al., 2022). This may result in developing MDR strains that are challenging to manage using currently available antibiotics.

*Citrobacter* spp. can acquire antibiotic-resistant determinants from related and non-related species. The existing carbapenemase gene repertoire in *Enterobacteriaceae* can mutate, evolve, and be transferred horizontally. The IncC plasmid-borne carbapenem-resistant determinants *bla_OXA-900_* are located in *C. freundii.* This gene is believed to have originated from a distinct Gram-negative bacterium *Shewanella*, a marine environmental extremophile (Frenk et al., 2021). In *C. koseri*, a single *bla_KPC-82_* (a bla_KPC-2_ variant) conferring resistance to β -lactam/β -lactam inhibitor combination was carried in a transposon integrated chromosomally. The transposon was initially harbored by a plasmid acquired by *C. koseri* from *S. marcescens* within the same host (Lebreton et al., 2021).

*Citrobacter* spp. are equipped with a repertoire of antibiotic efflux genes conferring intrinsic resistance and are highly efficient in horizontal gene transfer for acquired resistance within and outside its genus *Citrobacter*. Therefore, to identify the cause of antibiotic resistance in clinical settings, it is essential to look for common chromosomal mutations and include relevant plasmid-mediated antibiotic-resistant determinants for each class of antibiotics.

## Treatment

Various types of antibiotics, including monobactams, aminoglycosides, carbapenems, cephalosporins, sulfonamides, nitrofuran, chloramphenicol, quinolones, and colistin are used for the treatment of *Citrobacter* infections (Doran, 1999; McPherson et al., 2008; Samonis et al., 2009; Deveci and Coban, 2014; Hrbacek et al., 2020; Jiménez-Guerra et al., 2020; Chavan et al., 2021; Gogry et al., 2021). However, multidrug-resistant *Citrobacter* strains limit the use of empirical antibiotics. Various studies have found that lately, *Citrobacter* spp. are more sensitive to meropenem, imipenem, colistin, Tigecycline, piperacillin/tazobactam, and cefoperazone/sulbactam (Hawaldar and Sadhna, 2019; Lee et al., 2019; Yao et al., 2021). Therefore, those drugs might be a good option for treating *Citrobacter* infections. In addition, *In-vitro* studies have found that silver nanoparticles can be used to treat *Citrobacter* infections (Abady et al., 2021). Likewise, a recent study has found that phage-antibiotic combined treatment against *C. amalonaticus* indicated that a sublethal concentration of phages used as an adjuvant with antibiotics could be an effective therapeutic strategy (Manohar et al., 2022). Therefore, the combination of phages and antibiotics can be tested in the future on other types of *Citrobacter* spp., especially *C. freundii* and *C. koseri.*

## Conclusion

Infections caused by various MDR *Citrobacter* spp. are widespread, seriously threatening public health worldwide. The presence of virulence genes and prophages in the *Citrobacter* spp. accounts for its increased virulence in urinary, respiratory, and intra-gastrointestinal tract infections and other complications. Moreover, the emergence of MDR *Citrobacter* spp. resulted in difficult-to-treat infections in humans revealed, as reflected by the epidemiological studies. The information provided in this review paper will offer great benefits in addressing the health burden of *Citrobacter* spp.

## Author contributions

IJ: Writing – original draft, Writing – review & editing. SI: Writing – original draft, Writing – review & editing. AH: Writing – original draft. ZT: Writing – original draft. SS: Conceptualization, Writing – original draft, Writing – review & editing.
